# Study on Sintering Behavior, Heat and Wear Resistance of Refractory Metal Borides (HfB_2_, ZrB_2_) and Al-Ni Modified PDC

**DOI:** 10.3390/ma18225093

**Published:** 2025-11-09

**Authors:** Chuang Zhao, Wenhao Dai, Shaotao Xu, Baochang Liu

**Affiliations:** 1 State Key Laboratory of Deep Eearth Exploration and Imaging, College of Construction Engineering, Jilin University, Changchun 130026, China; zhaochuang21@mails.jlu.edu.cn; 2 Key Laboratory of Drilling and Exploitation Technology in Complex Conditions of Ministry of Natural Resources, Jilin University, Changchun 130026, China; 3 CNPC Greatwall Drilling Company, 101 Anli Road, Chaoyang District, Beijing 100020, China; daiwh18@mails.jlu.edu.cn; 4 School of Engineering and Technology, China University of Geosciences, Beiiing 100083, China; xust815@163.com; 5 State Key Laboratory of Superhard Materials, Jilin University, Changchun 130021, China

**Keywords:** polycrystalline diamond compact (PDC), refractory metal boride, coefficient of thermal expansion, vertical turret lathe (VTL) experiment

## Abstract

Polycrystalline Diamond Compacts (PDC) face thermal damage and insufficient wear resistance in complex strata due to the high thermal expansion coefficient of Co binder and its catalysis on diamond graphitization. Existing studies lack a systematic comparison of HfB_2_, ZrB_2_, and Al-Ni (1.5wt.%Al + 1.5wt.%Ni) on PDC performance under a unified process, and their synergistic mechanism with the PDC matrix remains unclear. Herein, 3wt.% of these additives were incorporated into diamond micropowder to prepare PDC via unified high-temperature and high-pressure (HTHP) sintering. XRD/SEM-EDS characterized the phase/microstructure, while thermal expansion and Vertical Turret Lathe (VTL) tests evaluated their properties. Results: (1) ZrB_2_-modified PDC performed the best, with a thermal failure temperature of 800 °C (8.5% higher than the blank group), VTL wear cycles of 110 Pass (22.2% higher), and ZrC (confirmed by XRD) enhancing interface bonding; (2) HfB_2_-modified PDC reduced the wear area by 18% (vs. the blank group) via low-expansion HfC (6.5 × 10^−6^/°C) and maintained a continuous structure; (3) Al-Ni-modified PDC had a wear ratio of 1.945 × 10^4^ (4.5% higher) but only 60 Pass and structural defects. This study confirms ZrB_2_ as the optimal additive for PDC’s comprehensive properties, supporting high-performance PDC development for complex downhole environments.

## 1. Introduction

Polycrystalline Diamond Compact (PDC) is fabricated by sintering diamond micropowder and metal binder on the surface of a cemented carbide substrate. Currently, cobalt (Co) is the most commonly used binder in industrial production. During the PDC sintering process, Co can promote the formation of D-D bonds between diamond particles, realizing the densification of diamond micropowder and thereby ensuring the structural integrity and mechanical properties of PDC [1,2]. However, the thermal expansion coefficient of Co is much higher than that of diamond, and Co tends to catalyze the graphitization transformation of diamond in high-temperature environments. These two defects cause thermal damage phenomena such as thermal stress cracking and strength attenuation in the polycrystalline layer of PDC during high-temperature operations. From the perspective of engineering application, PDC bits can efficiently break rocks under a load much lower than the rock strength when drilling in soft- to medium-hard formations and low-abrasiveness rock formations [3]. Nevertheless, in highly abrasive formations (e.g., quartz sandstone), ultra-compact rocks (e.g., granite), as well as soft-hard interbedded and fractured formations, the wear rate of PDC bits accelerates significantly, and the drilling efficiency drops sharply, which has become a core technical bottleneck restricting the application of PDC in complex formations [4]. Moreover, when PDC bits work in downhole high-temperature and high-pressure environments, the continuous friction between the polycrystalline layer and rocks causes a sharp increase in surface temperature, which further intensifies the graphitization of diamond and ultimately leads to a significant reduction in the service life of PDC [5,6,7].

Currently, the synthesis methods of PDC polycrystalline layers are mainly divided into three categories: (1) Binder-free synthesis method [8]: Nano-polycrystalline diamond is prepared using graphite or non-graphite carbon sources. Although it has excellent performance, it requires ultra-high pressure (14 GPa) and high temperature (1600–2000 °C), and the finished product has a small size, which makes it difficult to meet the needs of industrial-scale production. (2) Solvent-binder synthesis method [9]: Taking carbon solvents such as Co as binders, polycrystalline diamond with D-D bonding is formed by promoting the melting and recrystallization of diamond. This type of PDC has high hardness, high wear resistance, and high toughness, making it the mainstream industrial technology at present. (3) Intermediate-phase binder synthesis method [10,11]: Elements such as Ti and Si are used to react with carbon to generate carbides (e.g., TiC, SiC [12]) as the bonding phase, forming D-M-D type polycrystalline diamond, which has the prominent advantage of excellent heat resistance. Scholars at home and abroad have carried out extensive research on the performance enhancement of PDC: The Institute of Advanced Manufacturing Technologies in Poland prepared TiB_2_-modified PDC using a Bridgman device and found that a 5% TiB_2_ addition could enable PDC to maintain high hardness at 800 °C [13,14,15,16,17,18,19,20,21]; The Institute of High Pressure Physics in Russia [22,23,24] developed high-stability PDC with oxidation resistance at 1200 K using boron carbide as a binder; The Federal University of Rio Grande do Norte in Brazil confirmed that Nb-modified PDC could reduce intergranular fracture by forming NbC [25]; Research by Element Six showed that the heat-resistant temperature of ZrB_2_-modified PDC was 250 °C higher than that of the blank group, and its high-temperature wear resistance was better [26]. However, existing studies still have limitations: although carbonate binders can improve wear resistance, they increase the brittleness of PDC; Al-Co binders require specific high-pressure conditions [27,28,29], and no study has systematically compared the performance enhancement effects of three additives (HfB_2_, ZrB_2_, and Al-Ni) under a unified process.

Both HfB_2_ and ZrB_2_ belong to refractory metal borides and are typical ultra-high temperature ceramic materials (UHTCs). Their melting points are both above 3000 °C, and they have high hardness, high strength, and excellent chemical stability. During the PDC sintering process, the high-temperature and high-pressure environment can provide energy for the carbothermal reduction reaction between HfB_2_/ZrB_2_ and diamond carbon sources, ultimately generating high-performance carbides such as HfC and ZrC. These carbides can not only inhibit the graphitization catalytic effect of Co but also improve the thermal stability of PDC. The Al-Ni system can form intermetallic compounds (e.g., AlCo_x_Ni_1−x_) [30,31] with long-range, ordered crystal structures. Such compounds have high yield strength, high fracture toughness, and good heat resistance; existing studies have confirmed that the Young’s modulus of Al-Ni-modified PDC can reach 850 GPa, and its wear resistance is more than twice that of commercial PDC. Although the performance advantages of the three additives have been initially verified, there are still obvious research gaps: first, the synergistic strengthening mechanism between HfB_2_/ZrB_2_/Al-Ni and the PDC matrix is not yet clear; second, no horizontal comparison of the heat-resistant and wear-resistant strengthening effects of the three additives (all with 3wt.% addition) has been conducted under a unified sintering process, making it impossible to determine the optimal type of additive.

To fill the above research gaps, the research objectives of this study are clearly defined as follows:

1. To systematically compare the effects of three additives (HfB_2_, ZrB_2_, and Al-Ni) on the heat resistance (e.g., thermal failure temperature, linear thermal expansion coefficient) and wear resistance (e.g., VTL wear cycles, wear area, wear ratio) of PDC under a unified high-temperature and high-pressure (HTHP) sintering process, focusing on clarifying how different additives improve PDC’s mechanical properties;
2. To reveal the synergistic strengthening mechanism between the three additives and the PDC matrix, including phase evolution (e.g., formation of HfC/ZrC/AlCo_x_Ni_1-X_), element interaction, and interface bonding improvement;
3. To screen out the additive with the optimal comprehensive performance, thereby providing theoretical support for the development of high-performance PDC suitable for complex downhole environments (where high heat and wear resistance are required).

To achieve these objectives, PDC samples with 3wt.% of each additive were prepared via unified HTHP sintering. X-ray Diffraction (XRD) and Scanning Electron Microscopy–Energy-Dispersive Spectroscopy (SEM-EDS) were used to characterize phase composition and microstructure, while thermal expansion tests and Vertical Turret Lathe (VTL) experiments evaluated heat and wear resistance, respectively.

## 2. Materials and Methods

### 2.1. Sample Preparation

**Figure 1:**
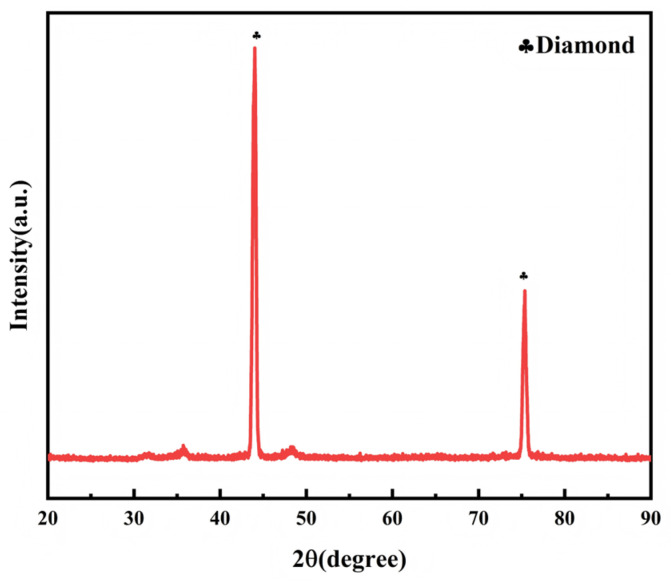
XRD pattern of W15-25 diamond micropowder.

The diamond micropowder used in this study was purchased from ILJIN Diamond Co., Ltd. (Seoul Special City, Republic of Korea), with the model of IMPN15-25 and a particle size distribution of 15–25 μm. Its XRD pattern is shown in Figure 1 [32]. A pyramid-grooved WC-Co-based cemented carbide containing 13wt.% Co was selected as the substrate in the experiment. Only the diamond micropowder with the above single particle size range was used, and no micropowder of other particle sizes was introduced. All additives used were elemental powders (without Co), and the Co element on the surface of the sintered PDC originated from the liquefaction and migration of the cemented carbide substrate at high temperature (i.e., the “sweeping effect”). In this experiment, a horizontal comparison was conducted using three types of additives, and the type, purity, and addition amount of the additives are detailed in Table 1.

**Figure 2:**
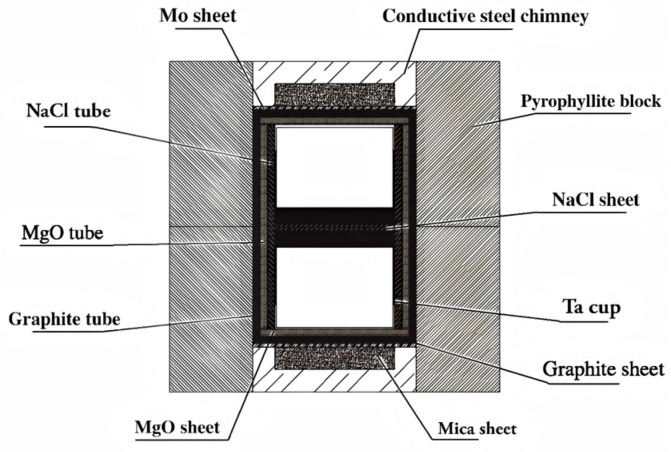
Schematic diagram of sintering cavity.

A planetary ball mill (manufacturer: LICHEN Technology Co., Ltd.; Shanghai, China) was used for the powder mixing process. Considering the high hardness of diamond powder, to avoid introducing impurities and contaminating the samples, WC balls with a diameter of 3 mm were selected as the grinding medium, and the ball-to-powder ratio was 10:1. During mixing, an alternating mode of “20 min of clockwise revolution + 20 min of counterclockwise revolution” was adopted, and the rotation speed of the mixing tank was set to 450 r/min. In the pre-installation stage, the mixed powder and the cemented carbide substrate were loaded together into a refractory metal cup, and then a 5 MPa hydraulic press (manufacturer: Huahui Hydraulic Technology Co., Ltd.; Dongguan, Guangdong, China) was used for pre-pressing the PDC green compact to ensure its structural densification and unify the height of the green compact (height difference ≤ 0.1 mm). All samples were heat-treated in the same Model ZGS-100 high-temperature vacuum heat treatment furnace (manufacturer: CETC 2nd Research Institute; sourced from Taiyuan, Shanxi, China) (800 °C, holding time for 12 h), and the schematic diagram of the sintering assembly is shown in Figure 2 [32].

A Type 650 hinge-type six-anvil press (model: CS-X(HD), manufacturer: Guilin Guiye Heavy Industry Co., Ltd.; sourced from Guilin, Guangxi, China) was used for sample sintering, and a conventional industrial basic sintering curve was adopted: the total heating time was 450 s (6.0 GPa,1550 °C), the oil pressure was constant during the pressure-holding stage, the heating power was fixed, and there was no acceleration or deceleration in the heating and cooling processes. After sintering, the samples were subjected to annealing treatment at 650 °C for 1 h. Finally, the samples were processed into conventional Type 1613 PDC (diameter: 15.9 mm, height: 13.2 mm) with a chamfer size of 0.38 mm, and the thickness of the polycrystalline diamond (PCD) layer was controlled to 2.2 mm.

### 2.2. Performance Testing Methods

This study mainly explores the influence of additive types on the heat resistance and wear resistance of PDC, and the testing methods are as follows:

#### 2.2.1. Heat Resistance Testing Method

Thermal expansion testing mainly relies on thermodynamic analysis (TMA) or a dedicated thermal dilatometer [21]. In this study, a PCY2000 Thermal Dilatometer manufactured by Xiangtan XIANGYI Instrument Co., Ltd. (Xiangtan City, Hubei Province, China) was used to conduct thermal expansion experiments on all experimental groups to determine the critical thermal stability temperature of the samples. The experiment was carried out by heating from 200 °C to 950 °C at a heating rate of 5 °C/min, and data were recorded every 12 s.

The linear thermal expansion coefficient is usually denoted by α, which defines the rate of dimensional change per unit length of a material when the temperature changes. The formula for the linear thermal expansion coefficient can be expressed as follows (1):

(1) $$\begin{array}{c} \alpha =\frac{∆L}{L\cdot ∆T} \end{array}$$

where α is the linear thermal expansion coefficient; ∆L is the change in length; L is the initial length; ∆T is the change in temperature.

When a material is heated in a high-temperature environment, the vibration of its molecules or atoms intensifies, leading to changes in the material’s structure and thus causing thermal expansion. The abrupt change point of the linear thermal expansion coefficient in the data curve is the heat resistance failure point of the material, which is usually located using the double tangent intersection method.

#### 2.2.2. Wear Resistance Testing Method

**Figure 3:**
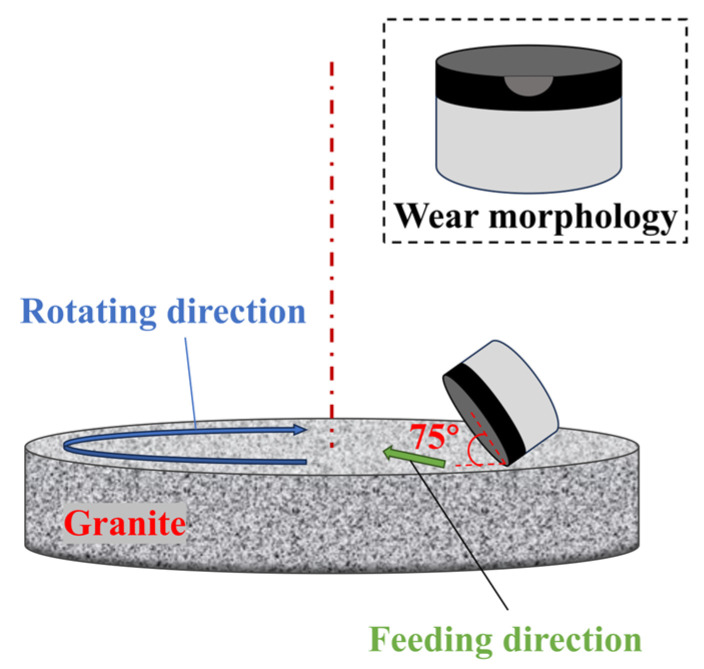
Schematic diagram of the VTL test principle.

At present, there are two most widely used methods for testing the wear resistance of PDC: one is Vertical Turret Lathe (VTL) grinding [33,34], and the other is diamond grinding wheel grinding. In this experiment, the VTL grinding method was adopted, which focuses on the determination of the wear ratio, and its test principle diagram is shown in Figure 3.

The ratio quantifies the relationship between tool wear and material removal efficiency by comparing the wear volume of the grinding head and the volume of the removed workpiece material (Formula (2)).

(2) $$\begin{array}{c} Wear\;Ratio=\frac{Tool\;Wear\;Amount\left(\mathrm{Wear}\;\mathrm{Volume}\right)}{Workpiece\;Removed\;Material\;Amount\left(\mathrm{Wear}\;\mathrm{Volume}\right)} \end{array}$$

The granite concentric disk used for wear testing has an outer diameter of 930 mm and an inner diameter of 270 mm. The samples were worn from the outermost radius to the inner radius, and the samples were non-polished; one sample was tested for each type of additive, with the VTL test bench (manufacturer: CHENGGONG Machine Tool Co., Ltd.; sourced from Quanzhou, Fujian, China) rotating at 101 rpm and the PDC sample moving from the edge to the center of the granite specimen at a speed of 5.08 mm/s; the traveling area per cycle was 0.62172 m^2^. By measuring the wear height of the granite per cycle, the amount of removed material in the wear ratio formula can be derived. The tool wear volume was calculated based on the wear area and height of the granite specimen abraded by the PDC. Definition of “Pass”: Each time the PDC specimen wears down the granite disk by a thickness of 0.5 mm, it is counted as 1 pass, and so on. The angle between the PDC and the granite was 15° (the angle between the PCD layer surface and the granite was 75°, and the angle between the cemented carbide substrate and the granite was 15°). As the number of wear passes increases, the wear area of the sample gradually increases. When the wear surface extends to the cemented carbide substrate, it is considered a sign of sample failure.

### 2.3. Characterization Results

**Figure 4:**
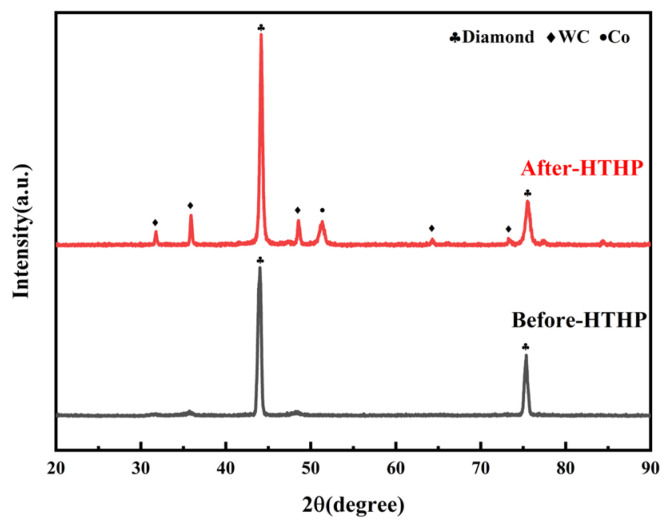
XRD patterns of the blank group before and after sintering.

**Figure 5:**
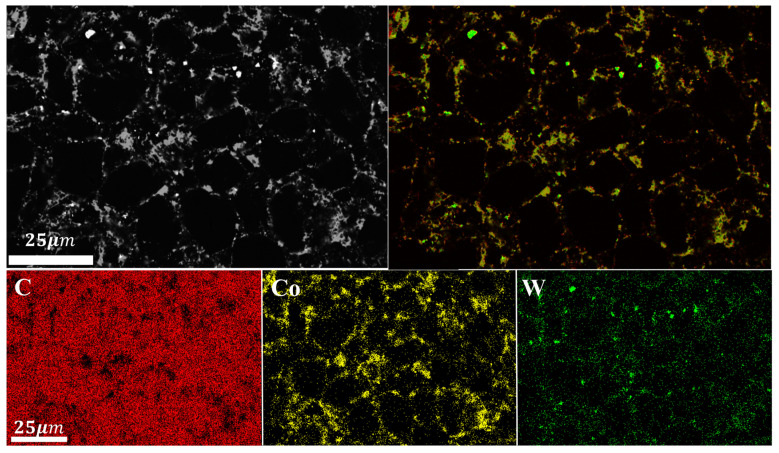
Surface EDS image of the blank group.

Through the analysis of the XRD patterns of the blank group (100wt.%DiaW15-25) before and after sintering (Figure 4 [32]), the sintering process of the polycrystalline layer can be roughly observed: WC and Co in the cemented carbide migrated into the polycrystalline layer. Additionally, a change in the diamond peak position was observed: the peak position at 2θ = 44° shifted to 2θ = 44.2°, and the peak position at 2θ = 75.36° shifted to 2θ = 75.46°, which indicates that high-temperature and high-pressure conditions can cause peak position shifts. The surface morphology of the polycrystalline layer of the blank group after sintering (Figure 5) shows that the main substances on the surface of the polycrystalline layer are diamond particles, WC, and Co pools. The Co pools are located in the gaps between diamond particles, which indicates that Co is absorbed by the van der Waals force between diamond particles (Table 2). During the observation of the surface morphology, a relatively bright phase was found, and subsequent EDS analysis of the surface confirmed that it was the W element. Not all gaps between diamond particles contain Co, which is because Co catalyzes the formation of D-D bonds between diamond particles. There were no gaps, height differences, or block shedding on the surface of the polycrystalline layer, and the sintering system was continuous.

**Figure 6:**
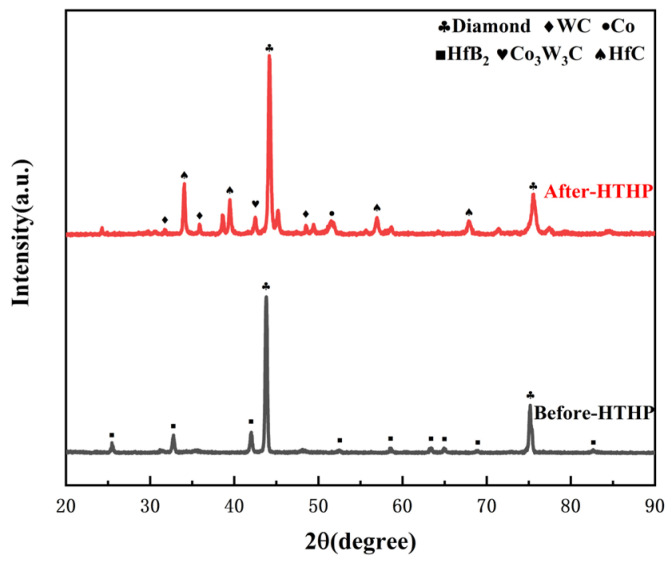
XRD patterns of the experimental group with HfB_2_ additive before and after sintering.

**Figure 7:**
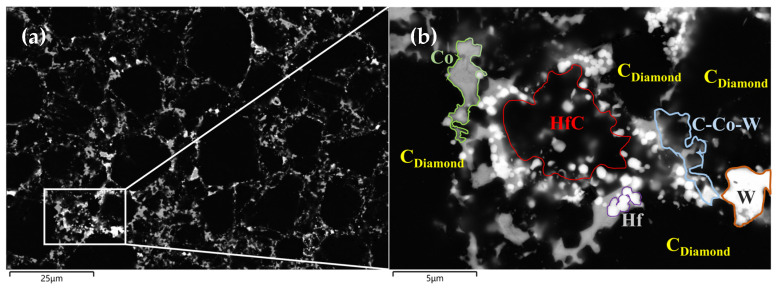
Surface SEM of the experimental group with HfB_2_ additive (**a**) and magnification of special area (**b**).

**Figure 8:**
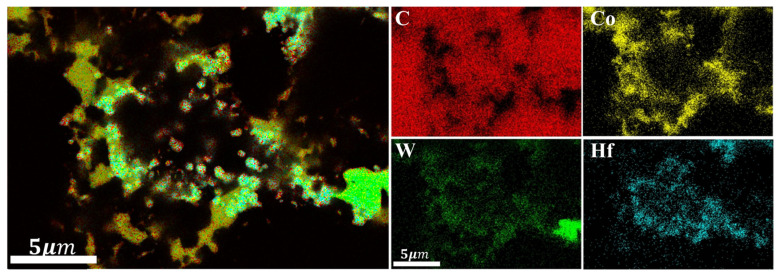
EDS elemental distribution of the magnified special area for the experimental group with HfB_2_ additive.

From the XRD patterns of the experimental group with 3wt.% HfB_2_-97.0wt.% DiaW15-25 before and after HTHP sintering (Figure 6 [32]), it can be seen that the WC balls and tanks used during powder mixing had a minimal overall impact on the powder. Before sintering, the sample mainly contained C (diamond) and HfB_2_. Before synthesis, the HfB_2_ peak (191) in the powder was very clear, but after high-temperature and high-pressure synthesis, the HfB_2_ peak disappeared, indicating that HfB_2_ decomposed during the synthesis process and new phases were formed. In the XRD pattern after sintering, HfC, HfO_2_, WC, and Co elements appeared, which reflect the phases of the product after sintering, and the pattern confirms the authenticity of the reaction described by the formula. During sintering, Co in the cemented carbide matrix melted and swept into the polycrystalline layer, carrying the W element in the matrix. A portion of HfB_2_ reacted with O elements in the system to form HfO_2_, and HfO_2_ further reacted with C elements in the system through a carbothermal reduction reaction to form HfC, a ceramic phase with more excellent wear resistance and heat resistance. In the EDS image (Figure 7b and Figure 8 and Table 3), in the area slightly to the right of the center (the elements are marked with color boxes in the figure), a large amount of W and Co elements were found through area scanning, and it is inferred that a W-Co-C solid solution phase may have been formed. By observing the EDS image data of the sample, large bulk HfC phases and strip-shaped HfC phases were formed between the diamond particles on the surface of the polycrystalline layer, among which the strip-shaped HfC phases mainly existed at the contact positions between diamond particles.

**Figure 9:**
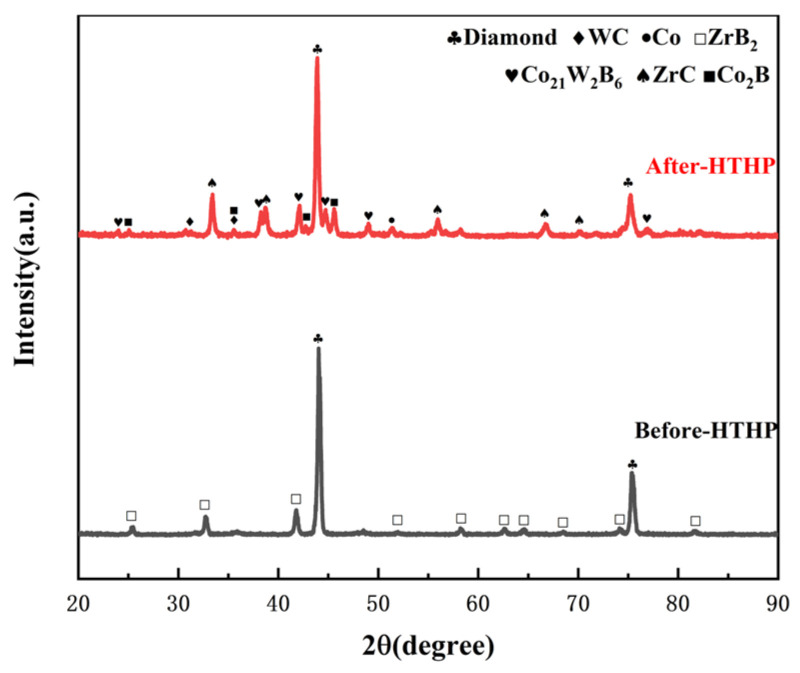
XRD patterns of the experimental group with ZrB_2_ additive before and after sintering.

**Figure 10:**
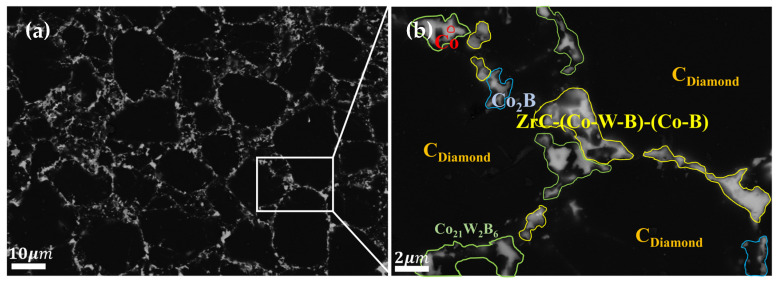
Surface SEM of the experimental group with ZrB_2_ additive (**a**) and magnification of special area (**b**).

**Figure 11:**
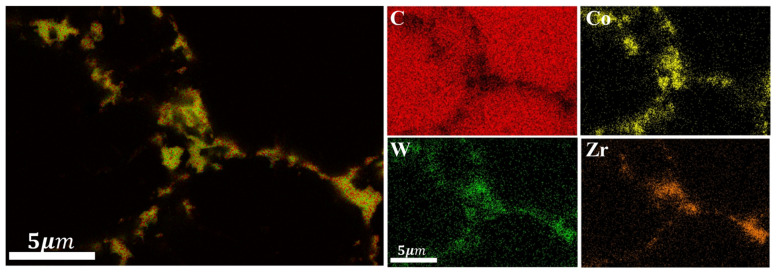
EDS elemental distribution of the magnified special area for the experimental group with ZrB_2_ additive.

XRD analysis was conducted on the polycrystalline layer of the 3.0wt.%ZrB_2_-97.0wt.%DiaW15-25 experimental group before and after sintering (the comparison of XRD patterns before and after sintering is shown in Figure 9 [32]). Before sintering, the main phases detected in the powder were ZrB_2_ and carbon (in the form of diamond), and no other impurity phases were detected, indicating that the original powder had high purity. The zirconium element in the ZrB_2_ additive reacted with the carbon source to form zirconium carbide (ZrC). This process was carried out through a carbothermal reduction reaction, in which the carbon element in the cemented carbide and the diamond (C Diamond) in the polycrystalline layer powder served as the carbon sources. This mechanism is similar to the mechanism by which HfC is generated in the HfB_2_ additive sample during high-temperature and high-pressure sintering. In the polycrystalline layer, cobalt (Co) existed not only in elemental form but also formed compounds containing cobalt, tungsten, and boron (Co_21_W_2_B_6_) and cobalt-boron compounds (Co_2_B). The EDS results (Figure 10 and Figure 11 and Table 4) show that the content of elemental Co in this additive sample system after sintering was 1.31wt.% lower than that in the system with HfB_2_ added, indicating that the conversion efficiency of elemental Co to Co-containing compounds was increased in this system. This can reduce the rate of reverse catalytic graphitization of D-D bonds by elemental Co at high temperatures and reduce the thermal damage of PDC.

**Figure 12:**
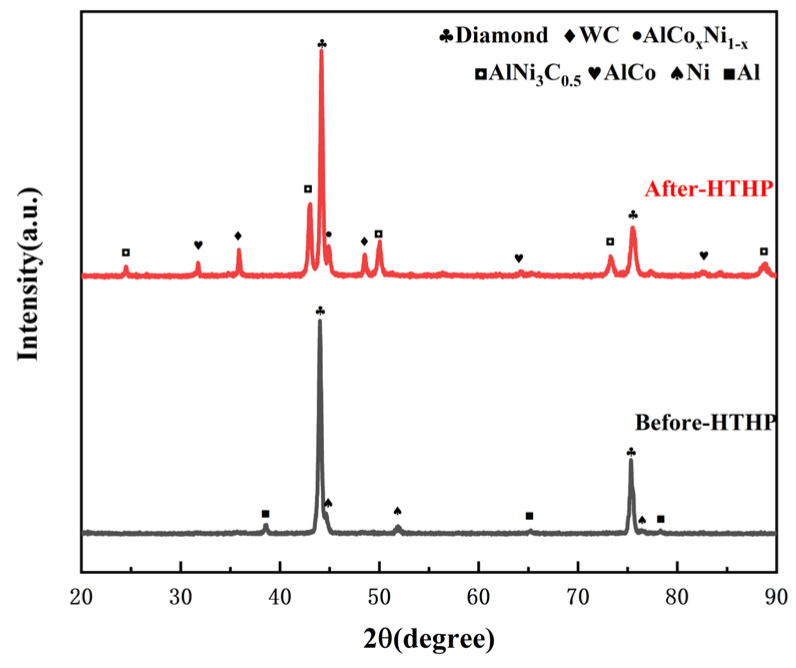
XRD patterns of the experimental group with Al-Ni additive before and after sintering.

**Figure 13:**
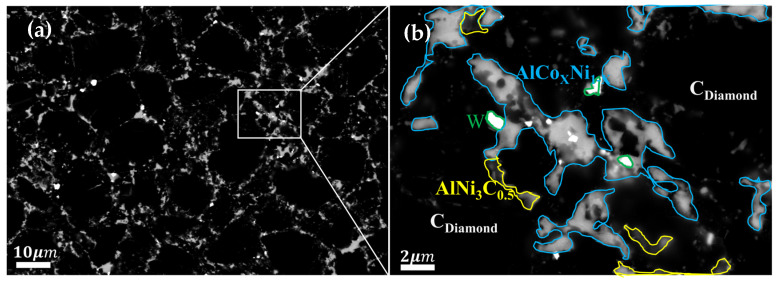
Surface SEM of the experimental group with Al-Ni additive (**a**) and magnification of special area (**b**).

**Figure 14:**
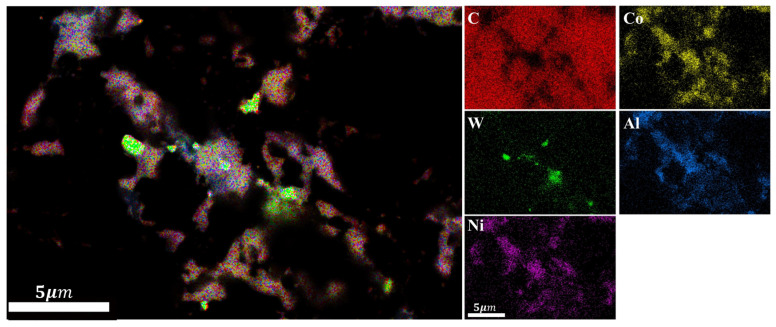
EDS elemental distribution of the magnified special area for the 1.5wt.% Al-1.5wt.% Ni experimental group.

XRD analysis was conducted on the polycrystalline layer of the 1.5wt.%Al-1.5wt.%Ni-97.0wt.%DiaW15-25 experimental group before and after sintering (Figure 12). Before high-temperature and high-pressure (HTHP) sintering, the XRD pattern showed the characteristic peaks of diamond, cemented carbide WC, and additives Al and Ni; after sintering, in addition to retaining the peaks of diamond and WC, new characteristic peaks of AlCo_x_Ni_1−x_ (x ≤ 1) and AlNi_3_C_0_._5_ composite phases appeared, and there were no peaks of pure Al or Ni. This indicates that Al and Ni almost completely reacted with Co and C. Among them, the peak position of AlCo_x_Ni_1−x_ proves that Al, Co, and Ni form a solid solution or compound, and the relative intensity and sharpness of the peak reflect its crystallization degree; the appearance of AlNi_3_C_0_._5_ confirms that Al and Ni react with Co and C simultaneously. SEM magnification and EDS element distribution analysis (Figure 13 and Figure 14 and Table 5) show that the content of AlCo_x_Ni_1−x_ in the mixed phase is significantly higher than that of AlNi_3_C_0_._5_. Al and Ni are the key elements for the formation of the two phases: AlCo_x_Ni_1−x_ is preferentially formed and has a high content, which may be due to the more sufficient interaction between Al and Ni under the catalysis of Co; in terms of distribution, AlCo_x_Ni_1−x_ is mostly formed in the regions enriched with Al and Ni (Al and Ni are uniformly distributed, so they exist in multiple regions), while AlNi_3_C_0_._5_ is only formed in the regions where C is locally enriched, with limited regions.

From the characterization results of the samples with various additives, all additives have been integrated into the respective systems and become part of the composite materials, with relatively continuous structures.

## 3. Results

### 3.1. Heat Resistance Testing

**Figure 15:**
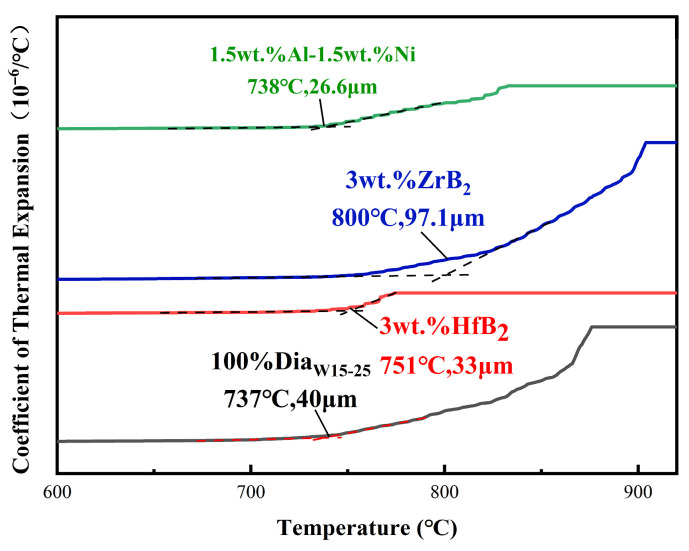
Linear thermal expansion coefficient of samples.

**Figure 16:**
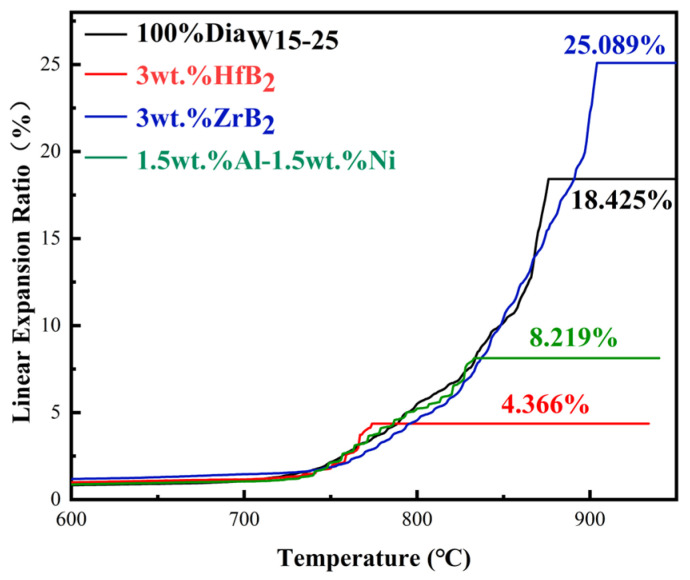
Axial linear expansion ratio of samples.

As shown in Figure 15 and Figure 16, there were significant differences in the thermal resistance failure temperature and the corresponding PDC axial linear expansion value among the samples of each group. The thermal resistance failure temperature of the blank group (without additive) was 737 °C, with an axial linear expansion value of 40 μm; the thermal resistance failure temperature of the 3wt.% HfB_2_ experimental group increased to 751 °C, and the linear expansion value decreased to 33 μm; the 3wt.% ZrB_2_ experimental group exhibited the optimal thermal resistance failure temperature, reaching 800 °C, but with a relatively high linear expansion value of 97.1 μm; the thermal resistance failure temperature of the 1.5wt.% Al-1.5wt.% Ni experimental group was 738 °C, which was basically the same as that of the blank group, and its linear expansion value was 26.6 μm.

Compared with the blank group, the experimental groups with HfB_2_ and ZrB_2_ added both showed better heat resistance. The heat-resistant temperature of the 3wt.% HfB_2_ experimental group was 14 °C higher than that of the blank group, with a 1.9% improvement in heat resistance. The 3wt.% ZrB_2_ experimental group had a more significant enhancement effect, with the heat-resistant temperature increasing by 63 °C and the heat resistance improving by 8.5%. This result confirms the feasibility that the refractory metal carbides (HfC, ZrC) generated during the sintering process can effectively enhance the heat resistance of PDC. These refractory carbides have strong high-temperature stability, which can inhibit the structural degradation of PDC at high temperatures and thus increase the thermal resistance failure temperature. However, the heat-resistant temperature of the 1.5wt.% Al-1.5wt.% Ni experimental group was basically the same as that of the blank group (737 °C, vs. 738 °C), indicating that under the synthesis process parameters of this experiment, this type of additive had no obvious improvement effect on the heat resistance of PDC and did not significantly change the high-temperature failure characteristics of PDC.

In addition to the thermal resistance failure temperature and volume expansion value, the system expansion rate, shown by the ordinate in the experimental figures (Figure 16), also exhibited obvious differences among groups. The final expansion rate data of each group are as follows: the blank group was 18.425%, the 3wt.% HfB_2_ experimental group was 4.366% (significantly lower than that of the blank group), the 3wt.% ZrB_2_ experimental group was 25.089% (higher than that of the blank group), and the 1.5wt.% Al-1.5wt.% Ni experimental group was 8.219% (lower than that of the blank group). The lower total expansion rate of the system with HfB_2_ added is mainly due to the difference in the linear thermal expansion coefficient of the phase composition: the linear thermal expansion coefficient of HfC generated after HfB_2_ sintering is 6.5 × 10^−6^/°C, which is much lower than that of the Co binder in PDC (13 × 10^−6^/°C). Under the same heating condition, the expansion capacity of HfC is much smaller than that of Co, which directly improves the structural stability of the polycrystalline layer. Moreover, HfC is exactly located at the contact interface between Co and diamond; when the polycrystalline layer expands due to heat, HfC can provide spatial buffering for the expansion of diamond and Co. Meanwhile, the unreacted HfB_2_ (linear thermal expansion coefficient: 6.0 × 10^−6^/°C) and the HfB (linear thermal expansion coefficient: 8.0 × 10^−6^/°C) generated by the reaction in the system both have lower linear thermal expansion coefficients than Co (13 × 10^−6^/°C) and C in diamond (8.5 × 10^−6^/°C), which further plays a role in buffering the expansion capacity.

The low expansion rate of the system with Al-Ni added is the result of the combined effect of the composition of the AlCoₓNi_1−x_ solid solution and the interface reaction: the value of x in AlCoₓNi_1−x_ ranges from 0 to 1 (when x = 1, it is AlCo; when x = 0, it is AlNi), and the linear thermal expansion coefficients of Al (23 × 10^−6^/°C, slightly increasing at high temperatures), Co (13 × 10^−6^/°C), and Ni (13.5 × 10^−6^/°C) differ significantly. The value of x (Co content) is the core variable: the larger the x value, the closer the expansion coefficient is to that of Co/Ni; the smaller the x value, the closer it is to that of Al. Finally, the expansion characteristics are regulated by the composition of the solid solution. At the same time, during the high-temperature and high-pressure reaction, AlCoₓNi_1−x_ may undergo an interface reaction with diamond (C) or WC, generating trace amounts of compounds such as Al-C (e.g., Al_4_C_3_, with a linear thermal expansion coefficient of only 5 × 10^−6^/°C) and W-Co. The linear thermal expansion coefficients of these impurity phases are significantly lower than those of metal phases, which further reduces the overall expansion coefficient of the system, ultimately resulting in a lower expansion rate.

### 3.2. Wear Resistance Testing

**Figure 17:**
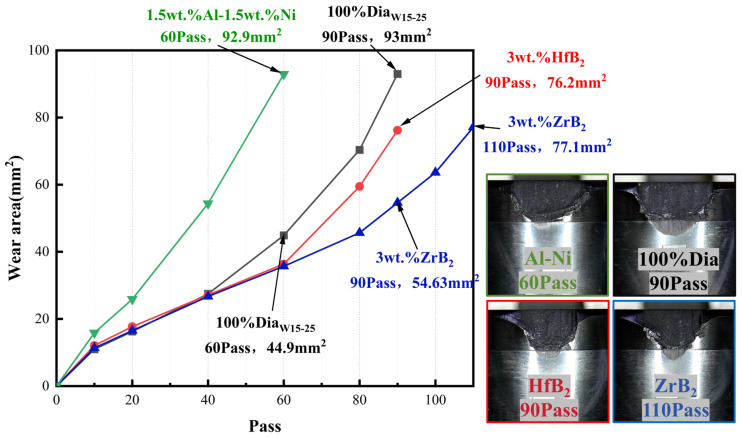
VTL test results of samples.

**Figure 18:**
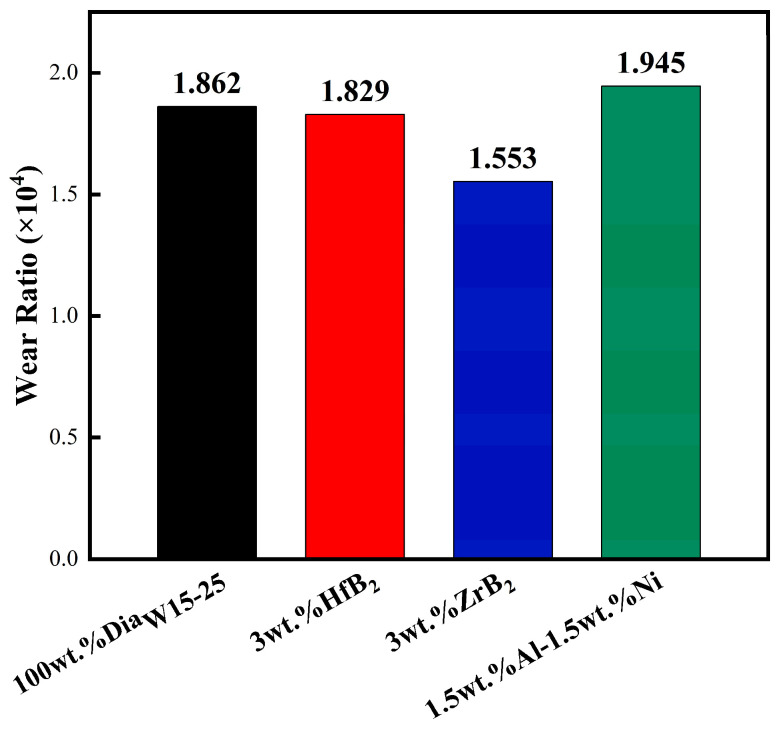
Wear ratio of samples.

The VTL test results can be analyzed from two aspects: first, the comparison of wear cycles (Pass). Under the same experimental conditions, the more wear cycles the PCD layer can withstand (Figure 17) and the smaller the wear area under the same number of cycles, the stronger the wear resistance of PDC; second, the wear ratio (Figure 18). A smaller value of this ratio indicates that more processing volume can be achieved when consuming the same volume of the grinding tool.

From the data of wear cycles, the blank group could work stably for 90 Pass; the experimental group with HfB_2_ added had the same maximum wear cycle count as the blank group, but the PCD wear port area was smaller, which was 18% less than that of the blank group. Combined with the wear ratio data (the HfB_2_ group was 1.829 × 10^4^, and the blank group was 1.862 × 10^4^, with similar values), it can be seen that the HfC and W-Co-B compounds generated in the PDC system with HfB_2_ added can improve the wear resistance of the system. In addition, the smaller wear area of the HfB_2_ group samples indicates that the low linear thermal expansion coefficient can effectively reduce the damage degree of the working surface, and there was no large chipping on the damaged surface, which further confirms that the internal structure of PCD is continuous and free of obvious defects.

The samples with ZrB_2_ added showed the highest number of wear cycles, which was 20 Pass higher than that of the blank group (with an increase of 22.2%). This phenomenon indicates that the ZrC generated during the sintering process is helpful to improve the bonding strength between different phases in PDC, thereby enhancing the overall connectivity of the material. The enhanced connectivity enables the composite material to better withstand external forces as a whole during the wear process and reduce the damage caused by local wear. Combined with the wear ratio data (the wear ratio of the ZrB_2_ group was 1.553 × 10^4^, a relatively small value), it can be seen that the PDC sintered with ZrB_2_ as the additive has a longer grinding life, but the grinding efficiency is relatively low.

The number of cycles until the samples with Al-Ni added were worn to failure was only 60 Pass, and the wear section area was equivalent to that of the blank group after 90 Pass. At the same time, signs of large chipping on the wear end face can be observed from the figure, indicating that the structure of this system was discontinuous under the sintering process parameters of this experiment, with weak structural surfaces. However, the wear ratio of this group was the highest among the four schemes (1.945 × 10^4^, 4.5% higher than that of the blank group), which shows that it is feasible to enhance the wear resistance of PDC by adding Al-Ni, but the powder processing and sintering processes need to be further optimized to improve the integrity of the internal structure of the material.

## 4. Conclusions

This study investigated the effects of HfB_2_, ZrB_2_, and Al-Ni additives on the properties of diamond compacts (PDC), focusing on the influence of additive types on the wear resistance and heat resistance of PDC under the same process conditions. The main conclusions are as follows:

1. For PDC with HfB_2_ addition, in addition to diamond, HfC phases were observed on the surface of the polycrystalline layer after sintering, and the content of HfC increased with the increase in the additive content. Large block-shaped HfC phases and strip-shaped HfC phases were formed between diamond particles on the surface of the polycrystalline layer. Additionally, Co-W-C phases were detected, all of which are phases that enhance mechanical properties. This system exhibited a smaller thermal expansion capacity, which is attributed to the low linear thermal expansion coefficient of the additive, thus providing a certain buffer for the thermal expansion of the system.
2. For PDC with ZrB_2_ addition, Co-W-C, Co-B, and ZrB phases appeared on the surface after sintering. This system could effectively reduce the content of elemental Co in the polycrystalline layer. The experimental group with ZrB_2_ addition achieved a test result of 110 working cycles, which was a 22.2% increase compared to the blank group. This study also confirmed the rule that reducing the content of elemental Co in the system can improve heat resistance.
3. For PDC with Al-Ni addition, intermetallic compounds AlCo_x_Ni_1−x_ and AlNi_3_C_0_._5_ phases were formed on the surface after sintering, which effectively reduced the content of elemental Co in the polycrystalline layer. PDC in this system had a relatively high wear ratio but a low number of wear cycles.

In summary, addressing the thermal damage and insufficient wear resistance bottlenecks of PDC in complex stratum applications, this study explored the regulation rules and mechanisms of 3wt.% HfB_2_, ZrB_2_, and Al-Ni on PDC’s phase composition and properties under a unified high-temperature and high-pressure (HTHP) sintering process. Results show that ZrB_2_ exhibits the optimal comprehensive performance by forming ZrC to enhance phase interface bonding, increasing PDC’s thermal resistance failure temperature to 800 °C and VTL wear cycles to 110 Pass; HfB_2_ effectively reduces the system’s thermal expansion rate and working surface damage by relying on HfC (linear expansion coefficient: 6.5 × 10^−6^/°C) generated by reaction, suitable for scenarios requiring low thermal deformation; although Al-Ni improves the wear ratio by forming AlCo_x_Ni_1−x_, the structural discontinuity caused by the current process needs optimization. Comprehensively, the 3wt.% ZrB_2_ additive group is recommended as the optimal choice for improving PDC performance in complex downhole environments, as it balances excellent heat resistance and wear resistance—two key properties for adapting to harsh downhole conditions. This study fills the gap in performance comparison of the three additives under a unified process, providing theoretical support for the composition design and process optimization of PDC for complex downhole environments.

## Figures and Tables

**Table 1:** Overview of experimental groups and additives.

Experimental Group	Type of Additive	Additive Content	Additive Particle Size
100wt.%DiaW15-25	/	0	/
3wt.%HfB_2_-97wt.%DiaW15-25	HfB_2_	3wt.%	2 μm
3wt.%ZrB_2_-97wt.%DiaW15-25	ZrB_2_	3wt.%	1 μm
1.5wt.%Al-1.5wt.%Ni-97wt.%DiaW15-25	Al	1.5wt.%	1 μm
	Ni	1.5wt.%	1 μm

**Table 2:** EDS mapping element content (wt.%) of the surface area shown in Figure 5.

Element Types	Surface Area
C	90.66
Co	7.74
W	1.60
Total Amount	100

**Table 3:** EDS mapping element content (wt.%) of surface area (shown in Figure 7a) and magnified special area (shown in Figure 7b).

Element Types	Surface Area	Special Area
C	89.64	81.05
Co	7.17	9.00
W	1.47	3.09
Hf	1.71	6.86
Total Amount	100	100

**Table 4:** EDS mapping element content (wt.%) of surface area (shown in Figure 10a) and magnified special area (shown in Figure 10b).

Element Types	Surface Area	Special Area
C	91.66	91.62
Co	5.86	4.39
W	1.02	0.92
Zr	1.46	2.08
Total Amount	100	100

**Table 5:** EDS mapping element content (wt.%) of surface area shown in Figure 13a and magnified special area shown in Figure 13b.

Element Types	Surface Area	Special Area
C	90.53	86.46
Co	5.71	7.59
W	0.65	1.00
Al	1.05	1.89
Ni	2.07	3.06
Total Amount	100	100

## Data Availability

The original contributions presented in this study are included in the article. Further inquiries can be directed to the corresponding author.
